# Select Per- and Polyfluoroalkyl Substances (PFAS) Induce Resistance to Carboplatin in Ovarian Cancer Cell Lines

**DOI:** 10.3390/ijms23095176

**Published:** 2022-05-05

**Authors:** Brittany P. Rickard, Xianming Tan, Suzanne E. Fenton, Imran Rizvi

**Affiliations:** 1 Curriculum in Toxicology & Environmental Medicine, University of North Carolina School of Medicine, University of North Carolina at Chapel Hill, Chapel Hill, NC 27599, USA; brickard@live.unc.edu (B.P.R.); suzanne.fenton@nih.gov (S.E.F.); 2 Department of Biostatistics, University of North Carolina School of Public Health, Chapel Hill, NC 27599, USA; xianming@email.unc.edu; 3 Lineberger Comprehensive Cancer Center, University of North Carolina School of Medicine, Chapel Hill, NC 27599, USA; 4 Division of the National Toxicology Program, National Institute of Environmental Health Sciences, Research Triangle Park, NC 27709, USA; 5 Center for Environmental Health and Susceptibility, University of North Carolina at Chapel Hill, Chapel Hill, NC 27599, USA; 6 Joint Department of Biomedical Engineering, University of North Carolina at Chapel Hill, Chapel Hill, NC 27599, USA; North Carolina State University, Raleigh, NC 27606, USA

**Keywords:** per- and polyfluoroalkyl substances, PFAS, ovarian cancer, platinum resistance, carboplatin, mitochondrial function

## Abstract

Per- and polyfluoroalkyl substances (PFAS) are ubiquitous environmental contaminants associated with adverse reproductive outcomes including reproductive cancers in women. PFAS can alter normal ovarian function, but the effects of PFAS on ovarian cancer progression and therapy response remain understudied. Ovarian cancer is the most lethal gynecologic malignancy, and a major barrier to effective treatment is resistance to platinum-based chemotherapy. Platinum resistance may arise from exposure to external stimuli such as environmental contaminants. This study evaluated PFAS and PFAS mixture exposures to two human ovarian cancer cell lines to evaluate the ability of PFAS exposure to affect survival fraction following treatment with carboplatin. This is the first study to demonstrate that, at sub-cytotoxic concentrations, select PFAS and PFAS mixtures increased survival fraction in ovarian cancer cells following carboplatin treatment, indicative of platinum resistance. A concomitant increase in mitochondrial membrane potential, measured by the JC-1 fluorescent probe, was observed in PFAS-exposed and PFAS + carboplatin-treated cells, suggesting a potential role for altered mitochondrial function that requires further investigation.

## 1. Introduction

Exposure to environmental contaminants may be a risk factor for a variety of diseases, including asthma, cardiovascular disease, and even cancer [1,2]. One class of environmental contaminants and endocrine-disrupting chemicals that have gained interest over the past decade are per- and polyfluoroalkyl substances (PFAS). PFAS are a class of widespread, persistent, and bioaccumulative chemicals that frequently pollute drinking water supplies worldwide [3,4,5,6,7,8,9]. These chemicals are also found in consumer products, including non-stick cookware, fast food packaging, wiring, cosmetics, and stain repellant furniture coatings, which constitute more minor routes of human exposure to PFAS [10,11,12,13].

Studies have shown that certain PFAS have adverse effects on a variety of bodily systems, including the endocrine, immune, and reproductive systems [14,15,16]. In the context of the female reproductive system, little is known about the effects of PFAS on the ovary. Limited studies have shown that certain PFAS may alter ovarian function [17,18]. Specifically, studies have implicated PFAS in impaired ovarian follicle formation, disruptions in steroid hormone levels, and reduced fertility [19,20,21,22,23,24]. Some studies have also found that certain PFAS are associated with increased risk or odds ratio of gynecologic disorders and diseases, including polycystic ovarian syndrome and even ovarian cancer at very high exposure levels; however, studies examining these associations are limited in sample size and/or number and warrant further investigation [18,25,26,27].

Ovarian cancer is the most lethal gynecologic malignancy, with a mortality rate of ~65% [28,29]. In 2022, approximately 19,880 women are estimated to be diagnosed with ovarian cancer, and 12,810 to succumb to the disease [28]. A major factor in the high lethality rate for ovarian cancer is resistance to therapy, specifically platinum-based chemotherapeutic agents [30,31]. Previous studies have shown that a broad range of factors may contribute to the development of platinum-resistant disease. Immune cells and cytokines in the tumor microenvironment, including those in ascites, or fluid accumulation in the abdomen, are associated with chemoresistant tumor cell populations [32,33,34,35,36,37]. Modulation of survival pathways and increased resistance to platinum-based therapy have also been demonstrated in ovarian cancer cells exposed to fluid shear stress [32,38,39,40]. In breast and liver cancer cell lines, exposure to environmental contaminants, including bisphenol A and hexabromocyclododecane, increases resistance to cisplatin, a platinum-based chemotherapeutic agent [41,42,43]. No prior studies have described the impact of PFAS exposure on treatment response in any cancer. The focus of the current report is to determine whether PFAS exposure contributes to platinum resistance in ovarian cancer.

PFAS frequently pollute drinking water supplies, and a hotspot of PFAS contamination is North Carolina [44]. In a recent study, three of the top PFAS contaminants in water supplied by the Orange Water and Sewer Authority, which services Chapel Hill and the surrounding area, were identified as perfluorooctanoic acid (PFOA), perfluoroheptanoic acid (PFHpA), and perfluoropentanoic acid (PFPA) [45]. Humans are rarely exposed to just an individual PFAS, and a mixtures approach may align best with human exposures.

NIH: OVCAR-3 (OVCAR-3) and Caov-3 are two human ovarian cancer cell lines that exhibit the molecular profiles representing high-grade serous carcinoma [46], the most common and lethal subtype of ovarian cancer [47,48,49,50]. Although these two cell lines are predicted to progress into the same tumor type, they may have slightly different bioenergetic modalities [51].

Previous studies have shown that PFAS affect mitochondrial function to cause adverse health outcomes in liver and other tissues [17,52,53,54,55,56,57]. Specifically, studies examining the mechanisms underlying the adverse effects of PFAS on ovarian biology have reported disruptions in the mitochondrial respiratory chain [17,52]. This is important because studies examining ovarian cancer bioenergetic profiles have also reported that, compared with platinum-sensitive cells, platinum-resistant ovarian cancer cells are highly metabolically active, meaning that these cells have increased flexibility in utilizing glycolysis and oxidative phosphorylation for energy production [51]. These findings implicate mitochondrial function, specifically metabolic activity, in platinum resistance. To determine whether mitochondrial function plays a role in PFAS-induced platinum resistance, mitochondrial membrane potential (ΔΨ_m_) was measured. ΔΨ_m_ can be measured using fluorescent cationic dyes such as 5,5′,6,6′-tetrachloro-1,1′,3,3′-tetraethylbenzimidazolylcarbocyanine iodide (JC-1) or Rhodamine 123, among others [58,59,60]. These cationic dyes have been shown to accumulate in hyperpolarized mitochondria, whereas less dye accumulates in depolarized mitochondria [58,59,60]. In this study, JC-1 dye was used to measure changes in ΔΨ_m_ following exposure to PFAS or PFAS mixtures and treatment with carboplatin.

In the present study, OVCAR-3 and Caov-3 cells were exposed to concentration ranges of PFOA, PFHpA, PFPA, or combinations of these agents. Survival fraction, defined as the fraction of viable cells normalized to the vehicle control, was measured after PFAS exposure, and two sub-cytotoxic concentrations were chosen for each chemical for future exposure experiments. PFAS-exposed ovarian cancer cells were then treated with a range of concentrations of carboplatin, a platinum-based chemotherapeutic agent commonly used to manage ovarian cancer [61,62]. Since mitochondrial dysfunction has been implicated after PFAS exposure and in platinum-resistant ovarian cancer cells, the relationship between PFAS exposure and mitochondrial function was explored in this study, and we hypothesized that PFAS might be disrupting mitochondrial function to increase survival fraction after carboplatin treatment. To explore this, ΔΨ_m_ was measured pre- and post-PFAS exposure in conjunction with carboplatin treatment. Findings from these experiments demonstrate that sub-cytotoxic exposures to certain PFAS increased the fraction of ovarian cancer cells surviving carboplatin treatment, indicative of resistance to chemotherapy, and that the mechanism underlying PFAS-induced resistance may be related to changes in mitochondrial function.

## 2. Results

### 2.1. Select Methanol Concentrations Decrease Survival Fraction in Ovarian Cancer Cell Lines

**Figure 1:**
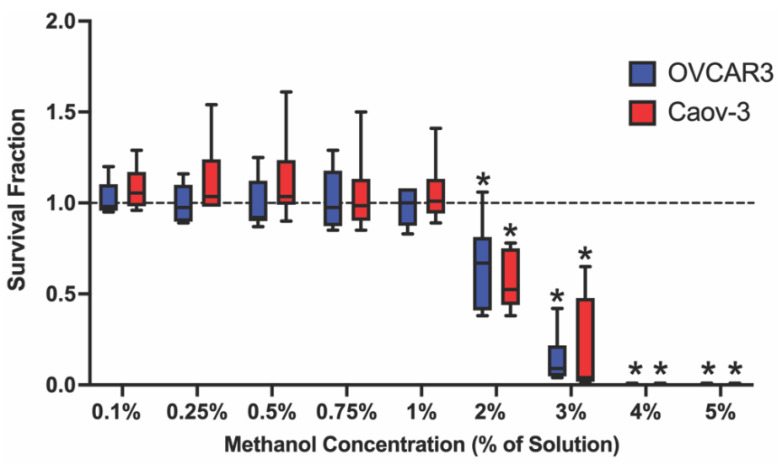
Concentration-dependent effect of methanol on survival fraction in two ovarian cancer cell lines. No significant reduction in survival fraction was observed in OVCAR-3 (blue) or Caov-3 (red) cells after 48 h incubation with ≤1% methanol (*v*/*v*) in complete cell culture medium (vehicle control for all subsequent experiments) (mean ± standard deviation (SD) expressed as a percentage of the no methanol control (dashed line); *n* = 3 independent experiments in duplicate). Significant differences between exposure group versus unexposed control are denoted by * (*p* < 0.05).

To ensure that methanol was not cytotoxic to each cell line, a concentration-range study of 0.1–5% methanol was performed. At concentrations greater than 1%, methanol significantly decreased survival fraction compared with controls (Figure 1). As a result, 1% methanol (OVCAR-3: 0.98 ± 0.10, Caov-3: 1.05 ± 0.18) was selected for the remainder of the experiments as the vehicle control.

#### 2.1.1. Selected PFAS Exposures Were Sub-Cytotoxic in OVCAR-3 and Caov-3 Cells

**Figure 2:**
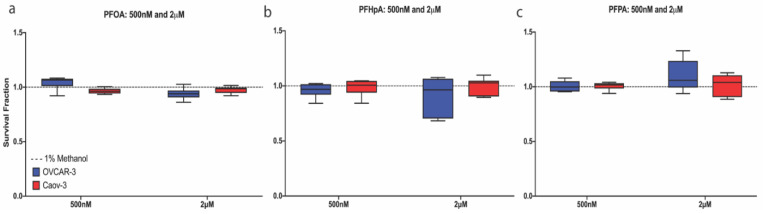
PFAS are sub-cytotoxic in ovarian cancer cell lines at selected nanomolar and micromolar concentrations. Relative to the vehicle control (dashed line), no significant difference in survival fraction was observed in OVCAR-3 (blue) or Caov-3 (red) cells after 48 h exposure to 500 nM or 2 μM (**a**) PFOA, (**b**) PFHpA, or (**c**) PFPA. Data are expressed as mean ± SD as a percentage of the vehicle control; *n* = 3 independent experiments in duplicate.

To examine the effects of nanomolar or micromolar concentrations of PFOA, PFHpA, or PFPA exposure on cell survival fraction, OVCAR-3 and Caov-3 cells were exposed to individual PFAS agents at concentrations ranging from 25 nM–2µM for 48 h (Figure S2a–c), including a 1-hour serum-free pulse (see Methods). Since PFAS are known to bind proteins in human serum, this serum-free pulse was performed to ensure adequate cell exposure to PFAS. Representative data in Figure 2 show that survival fraction in OVCAR-3 cells (blue) did not differ significantly from the vehicle control after exposure to 500 nM or 2 μM PFOA (1.041 ± 0.061 or 0.939 ± 0.053), PFHpA (0.96 ± 0.065 or 0.91 ± 0.172), or PFPA (1.005 ± 0.049 or 1.101 ± 0.142) (Figure 2a–c). Similarly, in Caov-3 cells (red), survival fraction was unaffected after exposure to 500 nM (0.965 ± 0.026) or 2 μM (0.976 ± 0.032) PFOA, 500 nM (0.986 ± 0.079) or 2 μM (0.997 ± 0.079) PFHpA, and 500 nM (1.007 ± 0.037) or 2 μM (1.017 ± 0.099) PFPA (Figure 2a–c). Since cytotoxicity was not observed after exposure to 500 nM or 2 μM PFOA, PFHpA, or PFPA in either OVCAR-3 or Caov-3 cells, these concentrations were used for subsequent experiments in both cell lines. A more extensive range of doses (25 nM–2 μM) was examined in the context of cytotoxicity, and results showed that in Caov-3 cells, all concentrations of PFAS that were tested were subcytotoxic. Interestingly, in OVCAR-3 cells, no cytotoxicity was observed at any concentration for PFOA or PFHpA; however, survival fraction significantly decreased compared with controls only after exposure to 100 nM PFPA (Figure S2a–c). All other PFPA concentrations tested were sub-cytotoxic in OVCAR-3 cells. These data demonstrate that at the majority of nanomolar and micromolar concentrations tested, PFOA, PFHpA, and PFPA do not affect survival fraction in OVCAR-3 and Caov-3 cells.

#### 2.1.2. PFAS Mixtures Increase OVCAR-3 and Caov-3 Cell Survival Fraction

**Figure 3:**
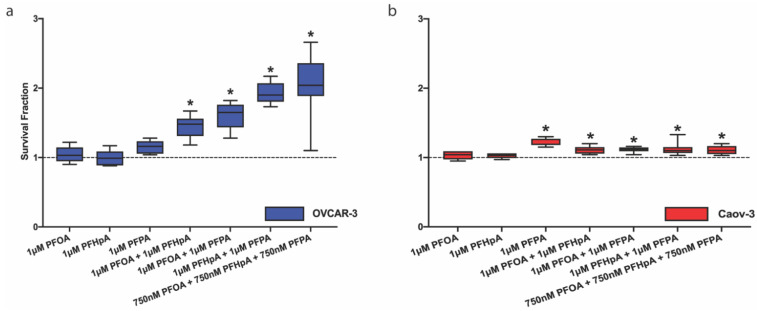
PFAS mixtures increase survival fraction in ovarian cancer cells. Exposure to PFOA + PFHpA, PFOA + PFPA, PFHpA + PFPA, or PFOA + PFHpA + PFPA for 48 h increases survival fraction in (**a**) OVCAR-3 (blue) and (**b**) Caov-3 (red) cells. Data shown are mean ± SD expressed as a percentage of vehicle control (dashed line); *n* = 3 independent experiments in duplicate for individual PFAS and *n* = 3 independent experiments in triplicate for mixtures. Significant differences between exposure group versus vehicle control are denoted by * (*p* < 0.05).

To build upon the human relevance of PFAS exposure, ovarian cancer cell lines were exposed to mixtures of PFOA, PFHpA, and/or PFPA. For these experiments, OVCAR-3 and Caov-3 cells were exposed to a 1:1 mixture of two individual PFAS or a 1:1:1 mixture of all three PFAS. Surprisingly, in both cell lines, PFAS mixtures increased survival fractions compared with controls. Figure 3a shows that survival fraction in OVCAR-3 cells increased after exposure to PFOA + PFHpA (1.440 ± 0.156), PFOA + PFPA (1.603 ± 0.187), PFHpA + PFPA (1.939 ± 0.156), and PFOA + PFHpA + PFPA (2.037 ± 0.444) compared with the vehicle control (1 ± 0.104). Similarly, survival fraction in Caov-3 cells increased after exposure to PFOA + PFHpA (1.107 ± 0.056), PFOA + PFPA (1.116 ± 0.037), PFHpA + PFPA (1.122 ± 0.089), and PFOA + PFHpA + PFPA (1.109 ± 0.063) compared with the vehicle control (1.001 ± 0.036) (Figure 3b). Additional doses of PFAS mixtures were also examined, and OVCAR-3 and Caov-3 cells both exhibited increased survival fractions post-low-dose PFAS mixture exposure (Figure S3a–h). In OVCAR-3 cells, these mixtures and concentrations included: 250 nM PFOA + 250 nM PFHpA (1.392 ± 0.199), 750 nM PFOA + 750 nM PFPA (1.810 ± 0.152), 250 nM PFHpA + 250 nM PFPA (1.614 ± 0.259), 500 nM PFHpA + 500 nM PFPA (1.914 ± 0.244), 750 nM PFHpA + 750 nM PFPA (1.864 ± 0.369), 100 nM PFOA + 100 nM PFHpA + 100 nM PFPA (1.803 ± 0.36), 250 nM PFOA + 250 nM PFHpA + 250 nM PFPA (2.22 ± 0.335), and 500 nM PFOA + 500 nM PFHpA + 500 nM PFPA (1.976 ± 0.241) (Figure S3a,c,e,g). In Caov-3 cells, these mixtures and concentrations included: 250 nM PFOA + 250 nM PFHpA (1.147 ± 0.063), 500 nM PFOA + 500 nM PFHpA (1.103 ± 0.035), 750 nM PFOA + 750 nM PFHpA (1.096 ± 0.077), 750 nM PFOA + 750 nM PFPA (1.15 ± 0.056), 500 nM PFHpA + 500 nM PFPA (1.082 ± 0.093), 750 nM PFHpA + 750 nM PFPA (1.116 ± 0.055), 250 nM PFOA + 250 nM PFHpA + 250 nM PFPA (1.119 ± 0.050), and 500 nM PFOA + 500 nM PFHpA + 500 nM PFPA (1.119 ± 0.037) (Figure S3b,d,f,h). Low-dose exposures to PFAS mixtures consistently increased OVCAR-3 and Caov-3 survival fractions across a variety of concentrations, suggesting proliferative effects of mixtures on ovarian cancer cells that warrant further examination.

### 2.2. At Baseline, Carboplatin Effectively Decreases Survival Fraction in Ovarian Cancer Cell Lines

**Figure 4:**
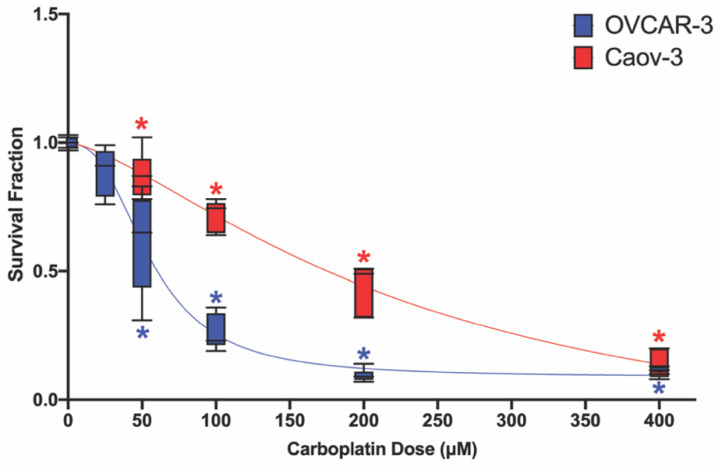
Dose–response to carboplatin in OVCAR-3 and Caov-3 cells. Dose-dependent reduction in survival fraction in OVCAR-3 (blue) and Caov-3 (red) cells following incubation with 0–400 μM carboplatin for 48 h. Data are shown as mean ± SD expressed as a percentage of the vehicle control (vehicle + 0 μM carboplatin); *n* = 3 independent experiments in duplicate. Curves were fitted using nonlinear regression analysis. Significant differences between treatment group versus vehicle control are denoted by * (*p* < 0.05).

As platinum-based chemotherapeutic agents are used in the standard of care for ovarian cancer treatment [61,62,63,64], this study explored the effectiveness of carboplatin, a platinum-based agent, in decreasing ovarian cancer cell survival fraction. Using a range of 25–400 μM carboplatin, concentration–response curves were established for both OVCAR-3 and Caov-3 cell lines. OVCAR-3 cells appeared to be more sensitive to carboplatin compared with Caov-3 cells, as 200 μM and 400 μM were both effective at reducing survival fraction by ~90% (0.095 ± 0.025 and 0.117 ± 0.012, respectively) compared with controls (Figure 4). Differences in sensitivity to carboplatin treatment between the two cell lines were further confirmed by the determination of IC_50_ values (OVCAR-3 = 60.61 μM carboplatin and Caov-3 = 175.8 μM carboplatin). In carboplatin-treated OVCAR-3 cells, 25 μM, 50 μM, and 100 μM reduced survival fraction by ~11% (0.888 ± 0.096), ~39% (0.613 ± 0.191), and 74% (0.26 ± 0.068), respectively (Figure 4). In Caov-3 cells, exposure to 50 μM, 100 μM, 200 μM, and 400 μM decreased survival fraction by ~12% (0.875 ± 0.088), ~28% (0.72 ± 0.06), ~56% (0.44 ± 0.094), and ~86% (0.138 ± 0.052), respectively (Figure 4). These data demonstrate that at baseline, carboplatin can be used to reduce survival fraction by over 85% in OVCAR-3 and Caov-3 cells.

### 2.3. PFAS Increase Survival Fraction Post-Carboplatin Treatment in Ovarian Cancer Cell Lines

**Figure 5:**
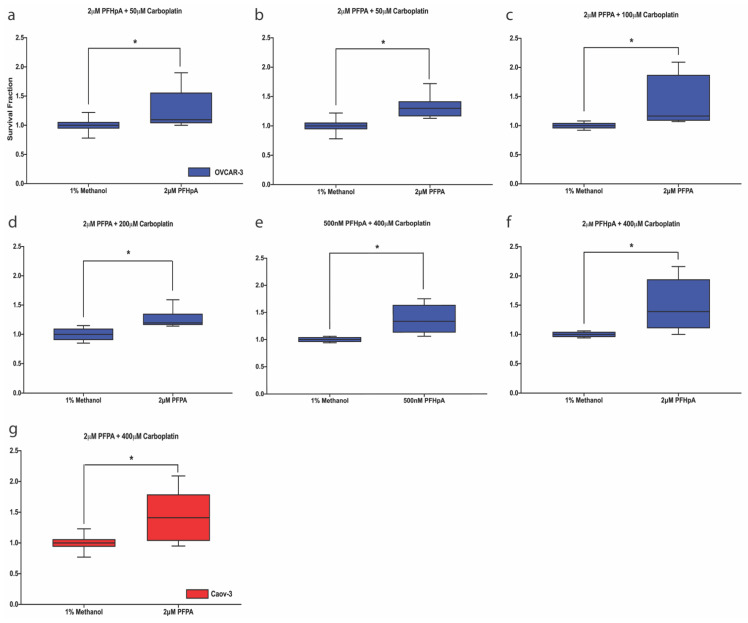
PFAS increase survival fraction in ovarian cancer cells treated with carboplatin. In OVCAR-3 cells (blue), increased survival fraction was observed after treatment with 50 μM carboplatin in (**a**) 2 μM PFHpA and (**b**) 2 μM PFPA-exposed cells, 100 μM carboplatin in (**c**) 2 μM PFPA-exposed cells, 200 μM carboplatin in (**d**) 2 μM PFPA-exposed cells, and 400 μM carboplatin in (**e**) 500 nM PFHpA and (**f**) 2 μM PFHpA-exposed cells, relative to vehicle controls. In Caov-3 cells (red), increased survival fraction was observed after treatment with 400 μM carboplatin in (**g**) 2 μM PFPA-exposed cells. Ovarian cancer survival fraction was assessed after 48 h exposure to PFHpA or PFPA followed by 48 h treatment with carboplatin. Data shown are mean ± SD expressed as a percentage of the vehicle control for each carboplatin group; *n* = 4 independent experiments in duplicate (OVCAR-3: 0, 25, 50, 100, and 200 μM carboplatin; Caov-3: all) or *n* = 3 independent experiments in duplicate (OVCAR-3 400 μM carboplatin). Significant differences between treatment group versus its own vehicle control are denoted by * (*p* < 0.05).

Since environmental contaminants have been shown to induce resistance to therapy in the context of other cancers [41,42,43], and data described above show that PFAS concentrations tested are sub-cytotoxic in ovarian cancer cell lines, the effect of PFAS on survival fraction post-carboplatin treatment was explored in OVCAR-3 and Caov-3 cells. Cells were exposed to either 500 nM or 2 μM of each PFAS followed by carboplatin treatment. After exposure to PFAS and carboplatin treatment, significant increases in survival fractions compared with the vehicle control were observed in both cell lines (Figure 5a–g). In OVCAR-3 cells, an increased survival fraction was observed in cells exposed to PFAS followed by treatment with 50–400 μM carboplatin, suggesting that these cells are more sensitive to PFAS exposure. For example, after exposure to PFAS and 50 μM carboplatin, survival fraction increased in OVCAR-3 cells exposed to 2 μM PFHpA (Figure 5a, 1.276 ± 0.333) or 2 μM PFPA (Figure 5b, 1.328 ± 0.194) compared with the vehicle control (1 ± 0.127). Cells exposed to 2 μM PFPA also exhibited increased survival fractions after exposure to 100 μM (Figure 5c, 1.395 ± 0.424) and 200 μM (Figure 5d, 1.264 ± 0.153) carboplatin. Additionally, OVCAR-3 cells exposed to 500 nM PFHpA (Figure 5e, 1.372 ± 0.278) and 2 μM PFHpA (Figure 5f, 1.492 ± 0.445) demonstrated increased survival fractions after exposure to 400 μM carboplatin compared with the vehicle control, indicative of carboplatin resistance. In Caov-3 cells exposed to PFAS and 400 μM carboplatin, 2 μM PFPA (Figure 5g, 1.441 ± 0.407) increased survival fraction compared with the vehicle control (1 ± 0.13). PFOA failed to cause a significant increase in survival fraction compared with the vehicle control after any carboplatin treatments, indicating that low doses of PFOA exposure do not affect carboplatin resistance in either cell line (Figure S4a,b). For clarity, IC_50_ values for OVCAR-3 and Caov-3 cells post-PFAS exposure and carboplatin treatment can be found in Table S1. Together, these findings demonstrate that upon exposure to select individual PFAS at sub-cytotoxic doses, OVCAR-3 and Caov-3 cells demonstrate increased survival fractions post-carboplatin treatment that were not observed at baseline.

**Figure 6:**
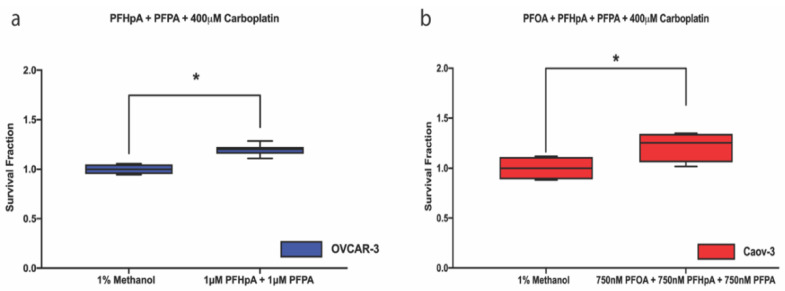
PFAS mixtures increase survival fraction following treatment with carboplatin in ovarian cancer cells. (**a**) Survival fraction in OVCAR-3 cells (blue) increases post-PFHpA + PFPA exposure and treatment with 400 μM carboplatin; and (**b**) in Caov-3 cells (red) post-PFOA + PFHpA + PFPA exposure and treatment with 400 μM carboplatin. Ovarian cancer survival fraction was measured after 48 h PFAS mixture exposure followed by 48 h treatment with carboplatin. Data are shown as mean ± SD expressed as a percentage of the vehicle control for each carboplatin group; *n* = 3 independent experiments in duplicate. Significant differences between treatment group versus its own vehicle control are denoted by * (*p* < 0.05).

To determine the effect of PFAS mixtures on carboplatin response, OVCAR-3 and Caov-3 cells were exposed to a 1:1 mixture of PFOA + PFHpA, PFOA + PFPA, or PFHpA + PFPA or a 1:1:1 mixture of PFOA + PFHpA + PFPA. Previous results (Figure S2a–h) suggest that all PFAS mixture concentrations tested were sub-cytotoxic; therefore, the highest concentrations of each mixture were selected for carboplatin response experiments. These experiments revealed that in both cell lines, increased survival fractions were observed at 400 μM carboplatin (Figure 6). In OVCAR-3 cells, exposure to 1 μM PFHpA + 1 μM PFPA (1.195 ± 0.057) increased survival fraction compared with the vehicle control (1 ± 0.053) (Figure 6a). In Caov-3 cells, exposure to 750 nM PFOA + 750 nM PFHpA + 750 nM PFPA (1.214 ± 0.140) increased survival fraction compared with the vehicle control (1 ± 0.111) (Figure 6b). While these were the only two instances of a significant increase in survival fraction following exposure to PFAS mixtures and treatment with carboplatin, exposure to other mixtures and carboplatin concentrations trended toward significance, warranting further exploration (Figure S5a,b). Overall, we demonstrated that PFAS alone, or as mixtures, increase survival fraction in ovarian cancer cell lines over a range of carboplatin concentrations (50–400 μM). IC_50_ values for OVCAR-3 and Caov-3 cells post-PFAS mixture exposure and carboplatin treatment can be found in Table S1. This is the first report associating PFAS exposure with increased survival fraction following treatment with platinum-based chemotherapy, indicative of platinum resistance, in the context of any cancer.

### 2.4. PFAS Alter ΔΨ_m_ in OVCAR-3 and Caov-3 Cells

**Figure 7:**
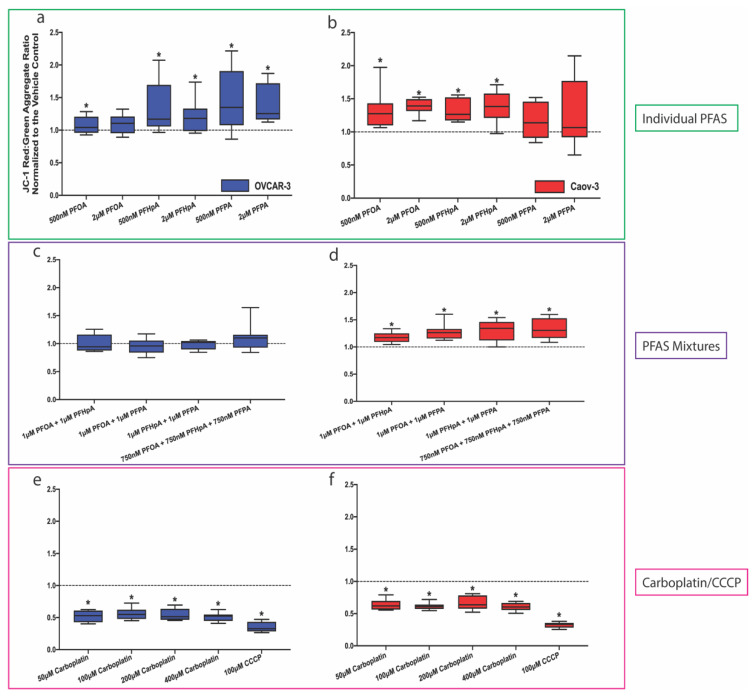
Exposure to certain PFAS led to an increase in ΔΨ_m_ while carboplatin treatment decreased ΔΨ_m_. PFAS (green box) increased ΔΨ_m_ in (**a**) OVCAR-3 and (**b**) Caov-3 cells. PFAS mixtures (purple box) did not affect ΔΨ_m_ in (**c**) OVCAR-3, but increased ΔΨ_m_ in (**d**) Caov-3 cells. Treatment with carboplatin or CCCP (pink box) decreased ΔΨ_m_ in (**e**) OVCAR-3 and (**f**) Caov-3 cells (mean ± SD expressed as a percentage of the vehicle control (dashed line); *n* = 4 independent experiments in duplicate). Significant differences between exposure group versus vehicle control are denoted by * (*p* < 0.05).

Since PFAS have been shown to alter mitochondrial function in the context of the ovary and other cell types [17,52,53,54,55,56,57], ΔΨ_m_ was evaluated in OVCAR-3 and Caov-3 cells following exposure to PFAS or PFAS mixtures and treatment with carboplatin (Figure 7). Twenty-four hours after seeding, OVCAR-3 and Caov-3 cells were exposed to 10 μg/mL JC-1 dye prior to a 1 h PFAS exposure and carboplatin treatment. Carbonyl cyanide m-chlorophenyl hydrazone (CCCP) was used as a positive control in these experiments. In OVCAR-3 cells, which appear to be more sensitive to PFAS exposure, significant increases in ΔΨ_m_ were observed after exposure to 500 nM PFOA (1.075 ± 0.132), 500 nM PFHpA (1.324 ± 0.407), 2 μM PFHpA (1.208 ± 0.256), 500 nM PFPA (1.474 ± 0.479), and 2 μM PFPA (1.386 ± 0.298) (Figure 7a). No significant alterations in ΔΨ_m_ were observed after exposure to PFAS mixtures (Figure 7c). Decreases in ΔΨ_m_ compared with controls were observed in OVCAR-3 cells treated with all doses of carboplatin (Figure 7e; 50 μM = 0.522 ± 0.093, 100 μM = 0.560 ± 0.090, 200 μM = 0.547 ± 0.092, 400 μM = 0.513 ± 0.068) and after CCCP treatment (0.344 ± 0.081). In Caov-3 cells, significant increases in ΔΨ_m_ were observed after exposure to 500 nM PFOA (1.324 ± 0.294), 2 μM PFOA (1.386 ± 0.120), 500 nM PFHpA (1.324 ± 0.168), and 2 μM PFHpA (1.372 ± 0.236), 1 μM PFOA + 1 μM PFHpA (1.174 ± 0.096), 1 μM PFOA + 1 μM PFPA (1.281 ± 0.150), 1 μM PFHpA + 1 μM PFPA (1.308 ± 0.191), and 1 μM PFOA + 1μM PFHpA + 1 μM PFPA (1.331 ± 0.200) (Figure 7b,d). Similar to OVCAR-3 cells, all doses of carboplatin (50 μM = 0.635 ± 0.082, 100 μM = 0.614 ± 0.053, 200 μM = 0.661 ± 0.106, 400 μM = 0.603 ± 0.063) and CCCP treatment (0.324 ± 0.043) led to significant decreases in ΔΨ_m_ (Figure 7f).

**Figure 8:**
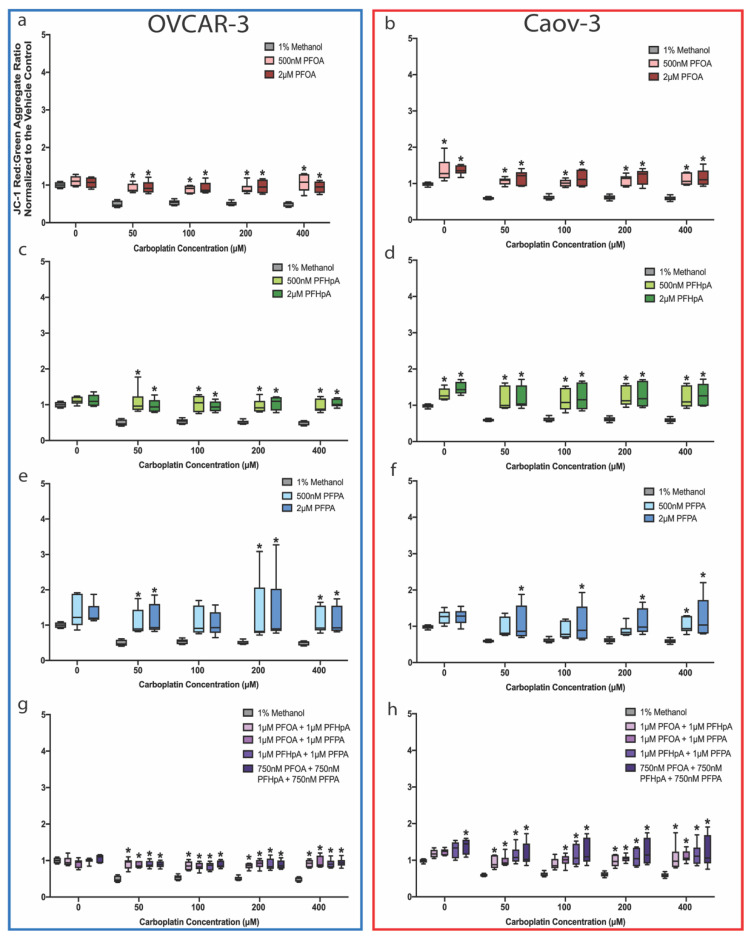
ΔΨ_m_ increases in OVCAR-3 and Caov-3 cells exposed to PFAS or PFAS mixtures then treated with carboplatin. ΔΨ_m_ increased compared to the vehicle control in OVCAR-3 (blue box) and Caov-3 (red box) cells after exposure to (**a**,**b**) PFOA, (**c**,**d**) PFHpA, (**e**,**f**) PFPA, or (**g**,**h**) PFAS mixtures at nearly all carboplatin concentrations examined (mean ± SD expressed as a percentage of the vehicle control; *n* = 3 independent experiments in duplicate). Significant differences between treatment group versus vehicle control are denoted by * (*p* < 0.05).

When cells were exposed to PFAS or PFAS mixtures then treated with carboplatin (Figure 8), increases in ΔΨ_m_ were observed in both cell lines for all compounds at nearly all concentrations of carboplatin (Figure 8, red, green, blue, and purple bars), compared with ΔΨ_m_ in carboplatin-treated cells that were not exposed to PFAS or PFAS mixtures (Figure 8, gray bars). For the sake of brevity, ΔΨ_m_ means ± SD are only highlighted for compounds and carboplatin concentrations at which platinum resistance was previously observed (Figure 5 and Figure 6). In OVCAR-3 cells, this includes 2 μM PFHpA + 50 μM carboplatin (0.971 ± 0.199), 2 μM PFPA + 50 μM carboplatin (1.154 ± 0.426), 2 μM PFPA + 100 μM carboplatin (1.033 ± 0.342), 2 μM PFPA + 200 μM carboplatin (1.383 ± 0.973), 500 nM PFHpA + 400 μM carboplatin (0.960 ± 0.184), 2 μM PFHpA + 400 μM carboplatin (1.042 ± 0.113), and 1 μM PFHpA + 1 μM PFPA + 400 μM carboplatin (0.916 ± 0.121) (Figure 8a,c,e,g). For Caov-3 cells, platinum resistance was only observed in the 2 μM PFPA + 400 μM carboplatin (1.239 ± 0.549) and 1 μM PFOA + 1 μM PFHpA + 1 μM PFPA + 400 μM carboplatin (1.227 ± 0.437) groups (Figure 8b,d,f,h). In each of these groups, ΔΨ_m_ significantly increased compared with the vehicle control at the corresponding carboplatin dose (OVCAR-3: 50 μM = 0.490 ± 0.086, 100 μM = 0.537 ± 0.071, 200 μM = 0.505 ± 0.058, and 400 μM = 0.495 ± 0.060; Caov-3: 50 μM = 0.595 ± 0.037, 100 μM = 0.611 ± 0.062, 200 μM = 0.612 ± 0.066, and 400 μM = 0.588 ± 0.064). It is important to note that the data in Figure 8 are internally normalized to their own vehicle control without carboplatin treatment; data illustrating JC-1 aggregate fluorescence ratios of PFAS or PFAS mixture exposure groups internally normalized to the corresponding carboplatin-exposed vehicle can be found in Figure S6. Interestingly, ΔΨ_m_ was also measured in OVCAR-3 and Caov-3 cells following 48-h PFAS exposure or carboplatin treatment followed by 48-h incubation with fresh medium, and significant decreases in ΔΨ_m_ were only observed following treatment with 200 μM (OVCAR-3 = 0.780 ± 0.182, Caov-3 = 0.865 ± 0.101) and 400 μM (OVCAR-3 = 0.715 ± 0.161, Caov-3 = 0.759 ± 0.102) carboplatin (Figure S7). These findings illustrate that ΔΨ_m_ is altered post-PFAS exposure and/or carboplatin treatment; thus, the role of mitochondrial function in ovarian cancer biology and response to chemotherapy warrants further investigation.

## 3. Discussion

Since therapy resistance is a major barrier to the effective treatment of ovarian cancer, this study sought to explore how exposure to environmental contaminants affects ovarian cancer cell response to carboplatin, a platinum-based chemotherapeutic agent. In the context of other cancers, including those of the liver and breast, environmental contaminants have been shown to induce resistance to chemotherapeutic agents [41,42,43]; however, the role of environmental contaminants in ovarian cancer therapy resistance has never been explored. Additionally, the role of PFAS in therapy resistance has not been examined in the context of any cancer. To address these knowledge gaps, this study explored the effects of select individual PFAS and PFAS mixtures on ovarian cancer survival fraction and response to carboplatin. Globally relevant PFAS were chosen, including the legacy molecule PFOA, and two less characterized and emerging compounds, PFHpA and PFPA. In addition to examining the effects of these individual PFAS, the effects of PFAS mixtures were also examined since mixtures are more human-relevant than individual PFAS alone [45,65,66].

Since certain PFAS and PFAS mixtures have been shown to induce toxicity across various cell types [14,15,16,67,68,69,70], this study first explored whether select nanomolar and micromolar concentrations of PFOA, PFHpA, and PFPA or mixtures of these agents were cytotoxic to ovarian cancer cells. Importantly, in comparison to the many studies examining the in vitro effects of PFAS, the concentrations used were markedly lower than those previously reported. Nanomolar and low micromolar concentrations (maximum = 2.25 μM) were intentionally chosen, as they are likely more relevant to human exposures compared with higher micromolar and millimolar concentrations. Rather than observing cytotoxicity, indicated by a decreased survival fraction compared with the vehicle control, PFAS mixtures increased survival fraction. While studies have previously reported proliferative effects of individual PFAS in human primary and transformed cell lines [71,72,73,74,75], this is the first study, to our knowledge, demonstrating an increase in survival fraction after exposure of ovarian cancer cells to PFAS mixtures. Despite individual PFAS having no significant effect on survival fraction, the finding that PFAS mixtures induce increased survival fraction is supported by prior publications. Previous studies examining the reproductive effects of individual antiandrogens, and mixtures of these agents, found that, even when individual compounds had no effect on an endpoint, antiandrogen mixtures led to cumulative, dose-additive effects [76,77,78]. Specifically, when separately evaluating in utero exposure to two androgen receptor antagonists—either vinclozolin or procymidone—Rider et al. [78] reported that vinclozolin alone induced hypospadias in 10% of male rats while procymidone alone had no effect male reproductive tract malformations (hypospadias or vaginal pouch development). When exposed to the two agents at those same doses as a mixture in utero, 96% of rats displayed hypospadias, and vaginal pouch development was observed in 54% of rats. Together, these findings suggest that mixtures of reproductive toxicants can produce dose-additive or synergistic effects in toxicity compared with individual agents. Findings in this current study warrant further investigation into the mechanisms underlying the PFAS mixture-induced increase in survival fraction.

While it is important to contextualize our in vitro results with PFAS doses used in vivo and those measured epidemiologically in human serum, reference doses for these compounds have yet to be established in the context of ovarian cancer. Studies measuring serum concentrations of PFAS in highly contaminated communities have reported that PFHpA serum concentrations ranged from 0.0001–0.0013 μg/mL, while PFOA serum concentrations ranged from 0.0017–0.011 μg/mL [79]. In the Veneto Region of Italy, maximum PFOA, PFHpA, and PFPA serum concentrations detected in the population were 1.4, 0.015, and 0.001 μg/mL, respectively [80]. Other studies evaluating PFOA serum concentrations in highly exposed communities have reported serum levels as high as 17.6 μg/mL [81]. While these values vary across studies, it is important to note that many factors may account for observed discrepancies, including geographic location, age, race, and other sociodemographic factors. Importantly, in the present study, the concentrations used of 25 nM–2 μM for PFOA, PFHpA, and PFPA correspond to 0.010–0.828 μg/mL, 0.009–0.728 μg/mL, and 0.007–0.528 μg/mL, respectively, illustrating that the concentrations used in this study are within range or within one order of magnitude of those reported in human biomonitoring studies.

Since the standard of care for ovarian cancer is a combination of platinum- and taxane-based chemotherapy [63,64], the effect of PFAS exposure on carboplatin response in ovarian cancer cells is critical to understand. While many ovarian cancer patients will effectively respond to this combination treatment initially, the majority develop recurrent disease that is often platinum resistant. The role of environmental contaminants in platinum resistance in ovarian cancer has never been explored but may prove critical for understanding how to better treat ovarian cancer patients with high levels of environmental exposure. To our knowledge, we are the first to report an association between ovarian cancer cell increased survival fraction post-environmental contaminant exposure and carboplatin treatment, indicative of platinum resistance. Specifically, increased survival fractions were observed in both OVCAR-3 and Caov-3 cell lines after exposure to both 500 nM and 2 μM concentrations of PFAS and carboplatin treatment at varying concentrations. Importantly, increased survival fraction post-carboplatin treatment was observed in ovarian cancer cells exposed to PFHpA or PFPA rather than PFOA. Compared with PFOA, which is very well studied, studies examining the effects of PFHpA and PFPA are limited [82], yet these two PFAS are found as contaminants in water systems across North Carolina [83] and numerous other states. These findings demonstrate the ability of PFHpA and PFPA exposure to induce adverse health effects, highlighting the need to investigate the potential health effects of these compounds further.

Select PFAS mixtures increased survival fractions post-carboplatin treatment in both OVCAR-3 and Caov-3 cell lines; however, significant differences compared with the vehicle control were only observed after exposure to one mixture in both cell lines. The lack of significance here may be due to variability in results, which is in accordance with previous studies from our lab (unpublished) demonstrating that external stressor exposure increases heterogeneity and, therefore, variability in the response of ovarian cancer cells. To further illustrate the effects of PFAS and PFAS mixtures on carboplatin dose–response, Table S1 contains IC_50_ values for each exposure group. Although some differences are observed across exposure groups, it is important to note that for many of these chemicals, resistance to platinum-based therapy was not observed until ovarian cancer cells were exposed to 400 μM carboplatin. As a result, certain PFAS and PFAS mixtures that induced resistance to carboplatin at 400 μM (at the end of the dose curve) do not appear to affect the IC_50_ value. Nonetheless, these findings demonstrate that certain PFAS increase ovarian cancer cell survival fraction post-carboplatin treatment, indicative of platinum resistance, and suggest that understanding environmental exposure profiles may be critical in understanding ovarian cancer patient responses to traditional chemotherapy.

Previous studies examining the underlying mechanisms by which PFAS lead to adverse outcomes have implicated disruptions in mitochondrial function [17,52,53,54,55]. Specifically, studies have noted that PFAS lead to adverse ovarian effects by disrupting the mitochondrial respiratory chain [17,52]. This is important because mitochondria have also been implicated in platinum resistance in ovarian cancer. Dar et al. [51] found that platinum-resistant ovarian cancer cells display a highly metabolically active phenotype, meaning that they have increased flexibility and capacity for using glycolysis and oxidative phosphorylation for energy production compared with platinum-sensitive cells, which primarily use glycolysis. Due to these existing associations between PFAS and mitochondrial disruption and platinum resistance and mitochondrial disruption, ΔΨ_m_, an indicator of cell health, was measured pre- and post-PFAS exposure and following carboplatin treatment. Under normal conditions, the interior of the mitochondria is negatively charged, promoting the inward movement of cations and outward movement of anions and creating an electrochemical gradient that drives adenosine triphosphate (ATP) synthesis [58,59,60]. Commercially, there are a variety of different dyes that can be used to measure ΔΨ_m_, each with their own set of benefits and limitations. For this study, the JC-1 dye was used to measure ΔΨ_m_ in PFAS-exposed and carboplatin-treated OVCAR-3 and Caov-3 cells. The JC-1 dye is slightly different from other commercially available dyes in that it forms aggregates, known as J aggregates, that accumulate in mitochondria and emit red fluorescence, compared with JC-1 molecules that emit green fluorescence [60]. The ratio of red to green fluorescence intensity can be used to evaluate changes in ΔΨ_m_. For example, compared to healthy cells, apoptotic cells will have a decreased red to green fluorescence ratio due to depolarization of the mitochondrial membrane that accompanies apoptosis [60]. Rather than observing a decrease in ΔΨ_m_, which has been previously reported in human liver cells, human lymphocytes, and osteoblast cells exposed to PFAS [54,57,84], exposure of OVCAR-3 and Caov-3 cells to individual PFAS agents resulted in an increase in ΔΨ_m_ in both cell lines. Interestingly, PFAS mixtures did not lead to similar increases in ΔΨ_m_ but also did not decrease ΔΨ_m_ significantly compared with controls. These findings differ from those following carboplatin treatment, which led to a decrease in ΔΨ_m_ at all concentrations tested. Previous studies evaluating the effect of platinum-based therapeutics on ΔΨ_m_ have reported contradictory findings. For example, Shen et al. [85] found that carboplatin decreased ΔΨ_m_ in HN-3 cells, a tongue squamous cell carcinoma line, and Chatterjee et al. [86] observed that the combination of sulforaphane with carboplatin decreased ΔΨ_m_ in human non-small-cell lung carcinoma cells. Conversely, Kleih et al. [87] found that cisplatin increased ΔΨ_m_ in OVCAR-3, OVCAR-4, and OVCAR-8 cells, although they also reported that cisplatin increased mitochondrial content in these cells.

Since PFAS induced platinum resistance in OVCAR-3 and Caov-3 cells, ΔΨ_m_ post-PFAS exposure and carboplatin treatment were also evaluated. In every exposure group (PFAS and PFAS mixtures), increased ΔΨ_m_ was observed for at least one carboplatin concentration in both OVCAR-3 and Caov-3 cells. These findings further confirm that PFAS exposure reduces cellular response to carboplatin, which, under normal conditions, decreased ΔΨ_m_ compared with controls. Previous studies have reported that in chemoresistant ovarian cancer cell populations, mitochondrial health is altered, as evidenced by increased bioenergetic flexibility between glycolysis and oxidative phosphorylation for energy production [51]. Other studies have shown that increases in ΔΨ_m_ confer survival advantages. For example, Grieco et al. found that malignant ovarian cancer cells with increased ΔΨ_m_ displayed enhanced autophagy, which promoted cell survival [88]. Additionally, a study evaluating the ability of ovarian cancer cells to adapt to hypoxic conditions found that supplementation with cysteine increased ΔΨ_m_ in OVCAR-3 cells under normoxic and hypoxic conditions, improving mitochondrial function and cell health [89].

Although dyes measuring ΔΨ_m_ can be beneficial for understanding cell health after exposure or treatment, these assays are not free from limitations. It is important to note that measuring changes in ΔΨ_m_ does not correspond to changes in the mitochondrial proton gradient ΔpH_m_ [58]. Additionally, the JC-1 probe, in particular, permeates slowly, is sensitive to loading concentrations and times, and is easily photobleached [58]. Since ΔΨ_m_ can be influenced by several factors, including changes in mitochondrial morphology, mass, or localization, it is important to verify findings using complimentary dyes and assays examining additional mitochondrial endpoints (ΔpH_m_, reactive oxygen species production, fission and fusion proteins, etc.) [58].

In summary, we report for the first time that at sub-cytotoxic concentrations, select PFAS and PFAS mixtures that are present in drinking water systems across the United States increase survival fraction of human ovarian cancer cells post-carboplatin treatment, indicative of carboplatin resistance. Increased survival fractions in ovarian cancer cells exposed to PFAS mixtures prior to carboplatin treatment were also noted. Results of carboplatin response studies also differed between cell lines, suggesting that while both cell lines are human epithelial ovarian adenocarcinoma lines, PFAS affect OVCAR-3 and Caov-3 cells differently. Specifically, OVCAR-3 cells appear to be more sensitive to PFAS exposure compared with Caov-3 cells, demonstrated by PFAS-induced increased survival fraction post-carboplatin treatment at lower concentrations of carboplatin. As a potential underlying mechanism for PFAS-induced increased survival fraction post-carboplatin treatment, ΔΨ_m_, an indicator of cell health, was measured. While significant increases in ΔΨ_m_ in OVCAR-3 and Caov-3 cells after exposure to select PFAS or PFAS mixtures and carboplatin were observed, further investigation is needed to understand the full effect of PFAS exposure on mitochondrial processes, specifically as it pertains to PFAS-induced platinum resistance.

## 4. Materials and Methods

### 4.1. Cell Culture

Human epithelial ovarian adenocarcinoma NIH:OVCAR-3 (OVCAR-3) cells and Caov-3 cells were obtained from The Physical Sciences-Oncology Network Bioresource Core Facility at American Type Culture Collection (ATCC). OVCAR-3 cells were grown in RPMI 1640 medium (Gibco, Thermo Fisher Scientific, Waltham, MA, USA) supplemented with 20% fetal bovine serum (FBS, Cytiva HyClone, Marlborough, MA, USA), 0.01 mg/mL bovine insulin (Sigma-Aldrich, St. Louis, MO, USA), 100 U/mL penicillin and 100 μg/mL streptomycin (Sigma-Aldrich). Caov-3 cells were grown in Dulbecco’s Modified Eagle’s Medium High Glucose (DMEM, Sigma-Aldrich) supplemented with 10% FBS, 100 U/mL penicillin, and 100μg/mL streptomycin. Cells used in this study were maintained in 2D monolayers at 37 °C in a humidified incubator with 5% CO_2_ and routinely tested for Mycoplasma contamination using the MycoAlert^TM^ PLUS Kit (Lonza Bioscience, Basel, Switzerland, Catalog #LT07-710). Cell stocks were replaced when the passage number exceeded 30. Both cell lines were authenticated by the Virology Core at the University of North Carolina at Chapel Hill using Ion Torrent Precision ID GlobalFiler^TM^ Next Generation Sequencing Short Team Repeat Panel (Applied Biosystems, Waltham, MA, USA).

### 4.2. Preparation of PFAS Stocks

Perfluorooctanoic acid (PFOA, CAS#335-67-1) in powder form was obtained from Synquest Laboratories (Alachua, FL, USA, Catalog #2121-3-18, 98% purity). Perfluoroheptanoic acid (PFHpA, CAS#375-85-9) in powder form was obtained from Sigma-Aldrich (Catalog #342041-5G, ≥97% purity). Perfluoropentanoic acid (PFPA, CAS#2706-90-3) in liquid form was obtained from TCI America (Portland, OR, USA, Catalog #N06055G, ≥98% purity). A total of 10mM stock solutions of each chemical was prepared in 100% potassium hydroxide and 1.0 N in methanol (referred to as “methanol” or “vehicle control”, Lab Chem Inc., Zelienople, PA, USA, Catalog #LC195402). Stock solutions were stored at −20 °C and allowed to equilibrate to room temperature prior to use.

### 4.3. Evaluation of Methanol Cytotoxicity

To determine the concentration of methanol in PFAS dosing solutions that could be applied to cells without causing cytotoxicity, OVCAR-3 and Caov-3 cells were seeded at a density of 5000 cells/well and 10,000 cells/well, respectively. Seeding densities were selected based on preliminary experiments exploring the linear dynamic range of the CellTiter Glo Assay for each cell line after 6 days (Figure S1). Twenty-four hours after seeding, cells were exposed to methanol in 50 uL serum-free media at concentrations ranging from 0–5% for 1 h. For OVCAR-3 cells, serum-free medium is RPMI 1640 supplemented with 100 U/mL penicillin and 100 μg/mL streptomycin. For Caov-3 cells, serum-free medium is DMEM High Glucose supplemented with 100 U/mL penicillin and 100 μg/mL streptomycin. A serum-free pulse is used for PFAS experiments, and this was performed to keep conditions between both experiments consistent. After 1 h, 0–5% methanol was added to 50 μL complete cell culture media for 47 h, for a total exposure time of 48 h (total media volume per well = 100 μL). Since carboplatin cytotoxicity experiments are 6 days in duration, methanol-containing medium was removed from cells on day 4 and replaced with fresh complete cell culture medium for 48 h. After a total of 144 h, survival fraction was measured using the CellTiter Glo Luminescent Cell Viability Assay (Promega Corp., Madison, WI, USA, Catalog #G7572). Briefly, plates containing cells were allowed to equilibrate to room temperature for 30 min prior to removing 50 μL medium from each well. Then, 50 μL of CellTiter Glo reagent was then added to each well, and plates were shaken orbitally for 2 min using the SpectraMax iD3 plate reader (Molecular Devices, LLC. San Jose, CA, USA). After 2 min, plates were removed from the platform, covered in foil, and the CellTiter Glo luminescent signal was allowed to stabilize for 10 min prior to performing the luminescence readout on the plate reader. While the CellTiter Glo protocol recommends using 100 uL of CellTiter Glo reagent, experiments comparing the readout strength of the CellTiter Glo assay when using 25 μL, 50 μL, or 100 μL of reagent showed minimal differences (Figure S1). Thus, 50 μL was chosen in an effort to minimize reagent used per experiment.

### 4.4. Evaluation of PFAS and PFAS Mixture Cytotoxicity

OVCAR-3 and Caov-3 cells were seeded at a density of 5000 cells/well and 10,000 cells/well, respectively. Cells were seeded in white-walled, clear-bottom 96-well plates and allowed to grow for 24 h prior to administering PFAS. On the day of exposure, PFAS solutions were prepared from stock solutions at concentrations ranging from 25–1000 µM. Individual PFAS agents were prepared at final concentrations ranging from 25 nM–2 µM (1% methanol) in 50 μL serum-free medium for 1 h. After 1 hour, individual PFAS agents at a final concentration ranging from 25 nM–2 µM in 1% methanol in 50 uL 2× serum medium were exposed for 47 h, for a total PFAS exposure time of 48 h. For OVCAR-3 cells, 2× serum medium was RPMI 1640 supplemented with 40% FBS, 0.02 mg/mL bovine insulin, 100 U/mL penicillin, and 100 μg/mL streptomycin. For Caov-3 cells, 2× serum medium was DMEM High Glucose supplemented with 20% FBS, 100 U/mL penicillin, and 100 μg/mL streptomycin. After PFAS exposure, medium was removed and replaced with 100 uL fresh cell culture medium. After 48 additional hours, survival fraction was measured using the CellTiter Glo Luminescent Cell Viability Assay and SpectraMax iD3 plate reader as previously described. For PFAS mixture cytotoxicity experiments, exposure solutions were prepared from stock solutions at concentrations ranging from 10–1000µM and were added at final concentrations of 2µM (2 PFAS mixture, 1 µM + 1 µM) or 2.25µM (3 PFAS mixture, 1 µM + 1 µM + 1 µM) in 1% methanol in 50 uL serum-free medium for 1 h. After 1 h, PFAS mixtures at final concentrations of 2 µM or 2.25 µM in 1% methanol in 50 μL complete cell culture medium were added for 47 h, for a total PFAS exposure time of 48 h. After 48 h of PFAS exposure, PFAS-containing medium was removed and replaced with 100 μL fresh cell culture medium. After 48 additional hours, survival fraction was measured as described previously.

### 4.5. Evaluation of Carboplatin Response Pre- and Post-PFAS Exposure

To measure the effects of PFAS on carboplatin response in human ovarian cancer cells, OVCAR-3 and Caov-3 cells were seeded in 96-well plates at densities of 5000 cells/well and 10,000 cells/well, respectively. Cells were allowed to grow for 24 h prior to PFAS exposure. PFAS solutions were prepared as described in Section 4.4. For these experiments, each PFAS agent was prepared at 500 nM or 2 µM, and these concentrations were chosen based on results of PFAS cytotoxicity experiments (Figure S2a–c). PFAS mixtures were prepared at final concentrations of 2 µM (2 PFAS mixture, 1 µM + 1 µM) or 2.25 µM (3 PFAS mixture, 750 nM + 750 nM + 750 nM) in 1% methanol. After a 1-hour serum-free PFAS pulse followed by a 47-h incubation with PFAS in 2× serum medium, PFAS-containing medium was removed, and fresh media containing 25–400 µM carboplatin was administered. Treatment solutions of carboplatin were prepared from the stock bottle (TCI, Catalog #C2043) by resuspending 1.8563 mg carboplatin in 1 mL medium to attain a 5 mM working solution. Using the 5 mM working solution, treatment solutions of 25 µM, 50 µM, 100 µM, 200 µM, and 400 µM were prepared in OVCAR-3 or Caov-3 medium. Carboplatin was added at the desired concentration in 100 μL media for 48 h prior to measuring survival fraction as previously described.

These steps were repeated without PFAS exposure for carboplatin dose–response experiments described in Section 2.2. To keep these experiments consistent with those involving PFAS exposure, PFAS-free serum-free medium containing 1% methanol was added to OVCAR-3 or Caov-3 cell cultures 24 h post-plating. After 1 h, PFAS-free 2X serum medium containing 1% methanol was added to OVCAR-3 and Caov-3 cell cultures for 47 h.

### 4.6. Evaluation of Ovarian Cancer Cell ΔΨ_m_ Post-PFAS Exposure

To measure the effect of PFAS on ΔΨ_m_, OVCAR-3 and Caov-3 cells were seeded in 96-well, black-wall, clear-bottom plates at a density of 40,000 cells/well. For signal optimization with the JC-1 dye (Invitrogen, Thermo Fisher Scientific, Catalog #T3168), cell densities of 10,000 cells/well, 20,000 cells/well, and 40,000 cells/well were used. The 40,000 cells/well routinely demonstrated the most consistent readouts and were, therefore, chosen for the remainder of experiments (data not shown). Cells were allowed to grow for 24 h prior to administration of 10 μg/mL JC-1 dye for 15 min. Exposure time and concentration of JC-1 dye were chosen based on previous findings using ovarian cancer cell lines with JC-1 dye [90]. After a 15 min incubation with 10 μg/mL JC-1 dye, cells were washed with phosphate-buffered saline (PBS) prior to exposure to individual PFAS agents (500 nM or 2µM PFOA, PFHpA, or PFPA), PFAS mixtures (2 PFAS mixture, 1 µM + 1 µM; 3 PFAS mixture, 750 nM + 750 nM + 750 nM), carboplatin (50 µM–400 µM), PFAS + carboplatin, PFAS mixtures + carboplatin, or 100 µM CCCP (Sigma-Aldrich, Catalog #215911-250 mg) for 1 h in a total of 100 uL serum-free (for PFAS and PFAS mixtures) or serum-containing (for carboplatin and CCCP) medium. Final concentration for CCCP was determined based on a pilot study demonstrating that compared with 50 µM or 75 µM, 100 µM CCCP had the most pronounced effect on ΔΨ_m_ when simultaneously incubated with JC-1 dye (data not shown). For individual PFAS, PFAS mixtures, carboplatin, and CCCP alone, exposures or treatments were performed in 100 uL serum-free or serum-containing medium containing 1% methanol. For combinations (PFAS + carboplatin and PFAS mixtures + carboplatin), PFAS/PFAS mixtures were added in 50 uL serum-free medium containing 1% methanol, and carboplatin was simultaneously added in 50 uL serum-containing medium containing 1% methanol. Pilot experiments with JC-1 dye evaluating optimal readout times revealed that readouts 1 h post-exposure and/or treatment, compared with immediate readouts or those performed 2 h post-exposure and/or treatment, had less varied and more consistent values (data not shown). The endpoint for this assay, JC-1 red:green aggregate ratio, was read using the SpectraMax iD3 fluorescence plate reader (green aggregate—excitation: 488 nm, emission: 529 nm; red aggregate—excitation: 488 nm, emission: 590 nm). Since JC-1 is a photosensitizer, all dosing was performed in the dark, and plates were covered with foil in lighted environments.

For consistency with other experimental timelines used in this study, ΔΨ_m_ was measured on experimental day 6, following 48-hours PFAS exposure + 48-h incubation with fresh medium or 48-hours incubation with 1% methanol + 48-h carboplatin treatment. For these experiments, OVCAR-3 and Caov-3 cells were seeded at 5000 cells/well and 10,000 cells/well respectively, and PFAS exposure and carboplatin treatment were performed as described in previous sections. On day 6, carboplatin-containing medium was removed, and medium containing 10 μg/mL JC-1 dye was added for 15 min. After 15 min, medium containing JC-1 dye was removed, and cells were washed with PBS prior to reading fluorescence using the method described above.

### 4.7. Statistical Analysis

To examine the effect of factors (e.g., PFAS concentration, carboplatin concentration) on outcomes of interest (e.g., survival fraction, mitochondrial membrane potential), unpaired *t*-tests, one-way or two-way ANOVA, or linear/nonlinear regression were employed, as appropriate. Specifically, unpaired *t*-tests were used to determine statistical significance in Figure 7, Figure 8 and Figure S6. One-way ANOVAs were run on data in Figure 1, Figure 2, Figure 3, Figure 4, Figures S2, S3 and S7. Appropriate contrasts from a two-way ANOVA were employed for Figure 5, Figure 6, Figures S4 and S5. To compare differences in outcomes between two groups (e.g., PFAS-exposed cells vs. vehicle control under a given carboplatin concentration), corresponding contrasts were extracted from linear regression analysis. Linear or nonlinear regression analyses were also performed in Figure 4 and Figure S1 to attain R^2^ values and fitted curves. All tests are 2-sided at alpha level 0.05 unless otherwise specified. All analyses were performed in R 4.1.1 [91] or Prism 9.0 software (GraphPad, San Diego, CA, USA). 

## Data Availability

Publicly available datasets were analyzed in this study. The data can be found here: https://manticore.niehs.nih.gov/cebssearch/paper/15444/private/F8EY4WJXYZ (accessed on 2 May 2022).

## Supplementary Materials

The following supporting information can be downloaded at: https://www.mdpi.com/article/10.3390/ijms23095176/s1.
