# Supplementary material for: Select Per- and Polyfluoroalkyl Substances (PFAS) Induce Resistance to Carboplatin in Ovarian Cancer Cell Lines
Source: Int J Mol Sci. 2022 May 5;23(9):5176. doi: 10.3390/ijms23095176 (PMC9104343; doi:10.3390/ijms23095176)
Supplement: Supplementary file 1 [file ijms-23-05176-s001.zip › ijms-1635030-supplementary_BPR.pdf]

Select Per- and Polyfluoroalkyl Substances (PFAS) Induce Resistance to Carboplatin in Ovarian Cancer Cell Lines

Brittany P. Rickard [1], Xianming Tan [2,3], Suzanne E. Fenton [1,4] and Imran Rizvi [1,3,5,6*]

[1] Curriculum in Toxicology & Environmental Medicine, University of North Carolina School of Medicine, University of North Carolina at Chapel Hill, Chapel Hill, NC 27599, USA; Brickard@live.unc.edu
[2] Department of Biostatistics, University of North Carolina School of Public Health, Chapel Hill, NC 27599, USA; xianming@email.unc.edu
[3] Lineberger Comprehensive Cancer Center, University of North Carolina School of Medicine, Chapel Hill, NC 27599, USA
[4] Division of the National Toxicology Program, National Institute of Environmental Health Sciences, Research Triangle Park, NC 27709, USA; suzanne.fenton@nih.gov
[5] Center for Environmental Health and Susceptibility, University of North Carolina at Chapel Hill, Chapel Hill, NC 27599, USA
[6] Joint Department of Biomedical Engineering, University of North Carolina at Chapel Hill, Chapel Hill, NC, and North Carolina State University, Raleigh, NC 27599, USA
* Correspondence: imran.rizvi@unc.edu

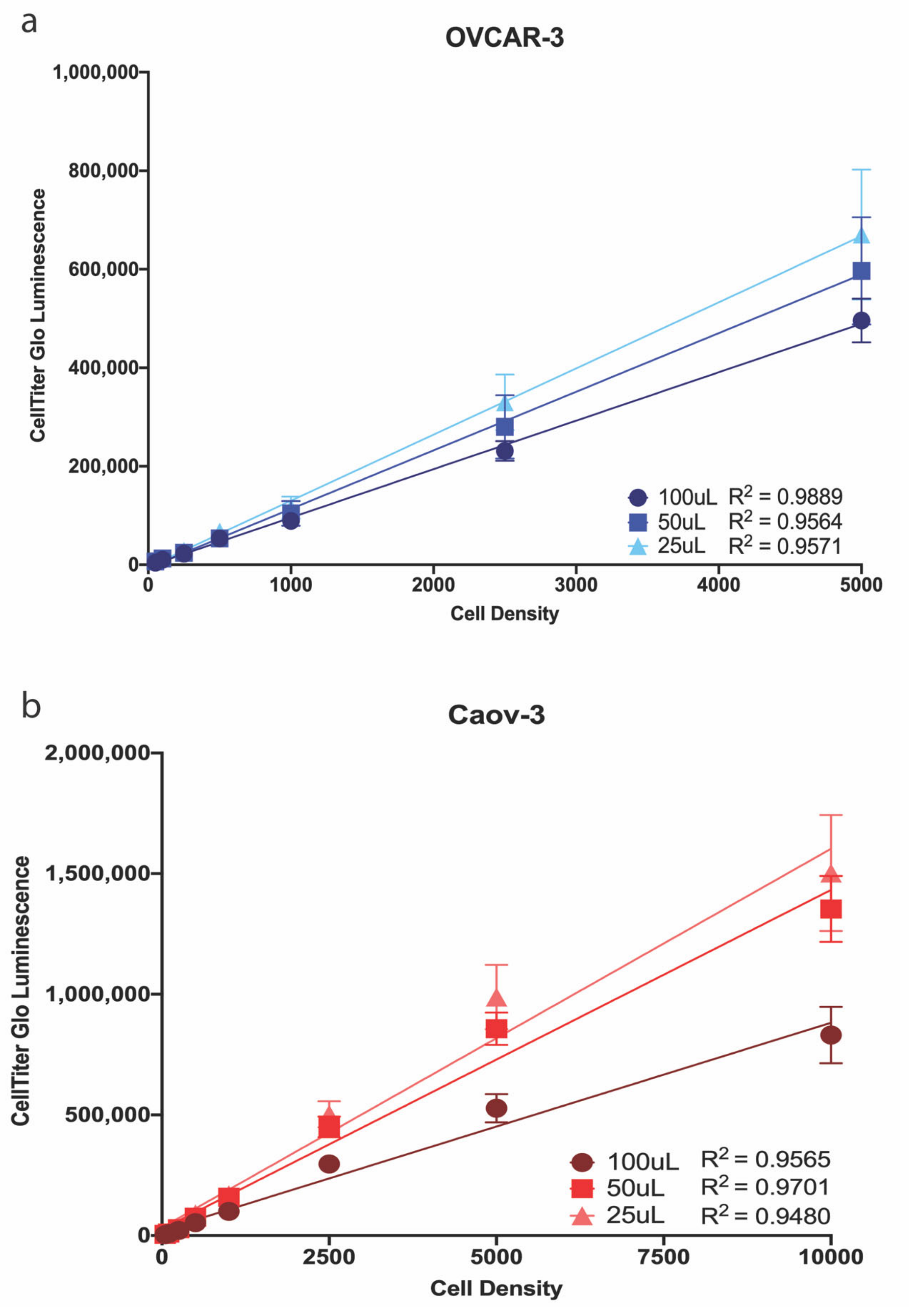

Figure S1. OVCAR-3 and Caov-3 optimal seeding densities and reagent volume based on CellTiter Glo linear dynamic range. CellTiter Glo luminescence readouts when **(a)** OVCAR-3 and **(b)** Caov-3 were plated at cell densities ranging from 0 – 5,000 cells/well and 0 – 10,000 cells/well, respectively, with the addition of 25, 50, or 100uL CellTiter Glo reagent [mean ± SD; *n* = 3 independent experiments in duplicate] after 6 days.

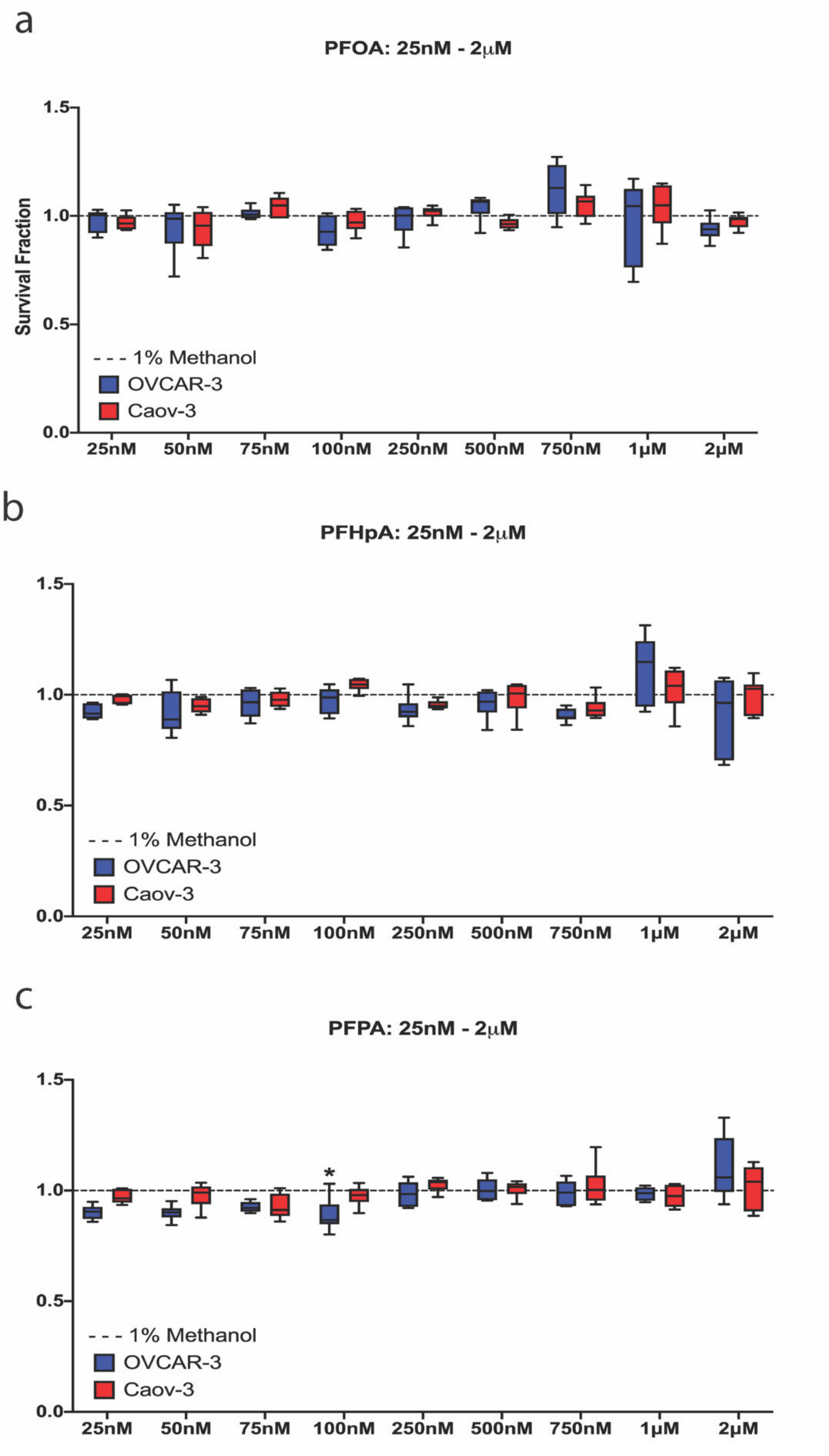

Figure S2. PFAS are mostly sub-cytotoxic in ovarian cancer cell lines at dose-ranging nanomolar and micromolar concentrations. OVCAR-3 (blue) and Caov-3 (red) survival fraction [mean ± SD expressed as a percentage of the vehicle control (dashed line); *n* = 3 independent experiments in duplicate] after 48 h exposure to 25nM - 2μM **(a)** PFOA, **(b)** PFHpA, or **(c)** PFPA.

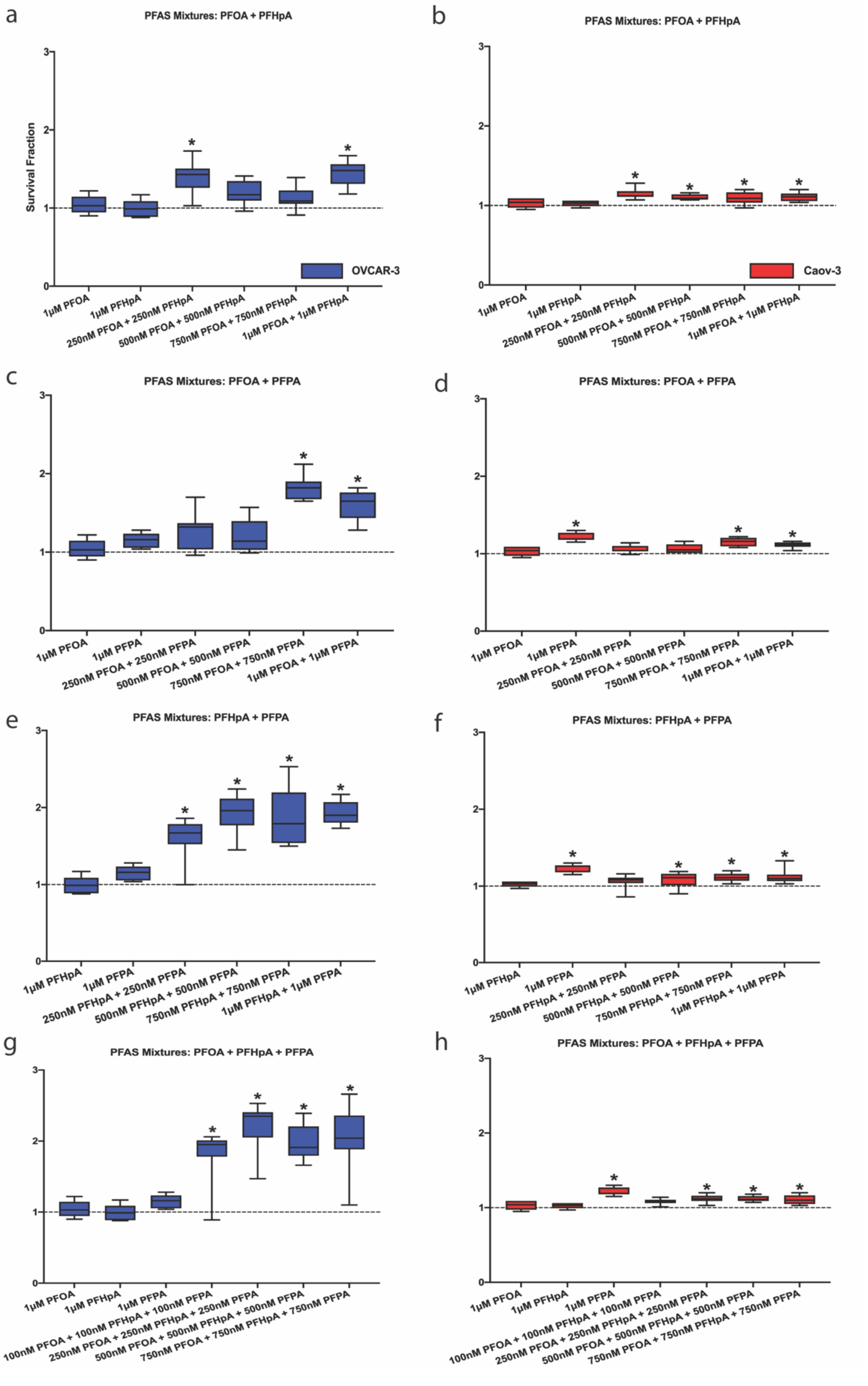

Figure S3. PFAS mixtures increase survival fraction in ovarian cancer cells. OVCAR-3 (blue) and Caov-3 (red) survival fraction [mean ± SD expressed as a percentage of the vehicle control (dashed line); n = 3 independent experiments in duplicate for individual PFAS, n=3 independent experiments in triplicate for mixtures] after 48 h exposure to **(a,b)** PFOA + PFHpA, **(c,d)** PFOA + PFPA, **(e,f)** PFHpA + PFPA, or **(g,h)** PFOA + PFHpA + PFPA.

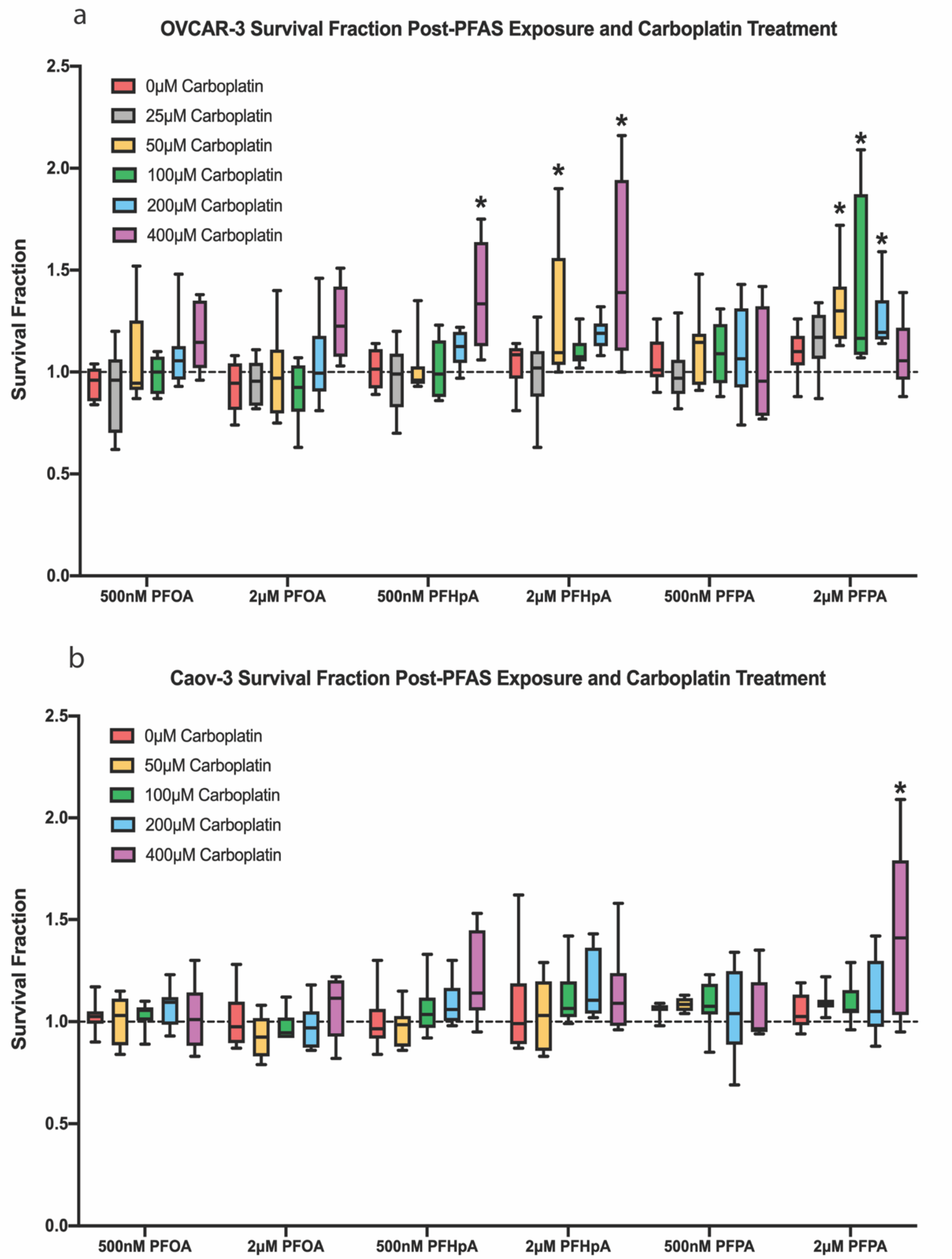

Figure S4. PFAS increase survival fraction post-carboplatin treatment in ovarian cancer cells. Select PFAS induce carboplatin resistance, indicated by an increase in survival fraction post-PFAS exposure and carboplatin treatment compared to the vehicle control (dashed line), in **(a)** OVCAR-3 and **(b)** Caov-3 cells. OVCAR-3 cell viability [mean ± SD expressed as a percentage of the vehicle control for each carboplatin group; $n = 4$ independent experiments in duplicate (0, 25, 50, 100, and 200μM carboplatin) or n=3 independent experiments in duplicate (400μM carboplatin)] after 48 h exposure to PFOA, PFHpA, or PFPA followed by 48 h treatment with carboplatin. Caov-3 viability [mean ± SD expressed as a percentage of the vehicle control for each carboplatin group; $n = 4$ independent experiments in duplicate] after 48 h exposure to PFOA, PFHpA, or PFPA followed by 48 h treatment with carboplatin.

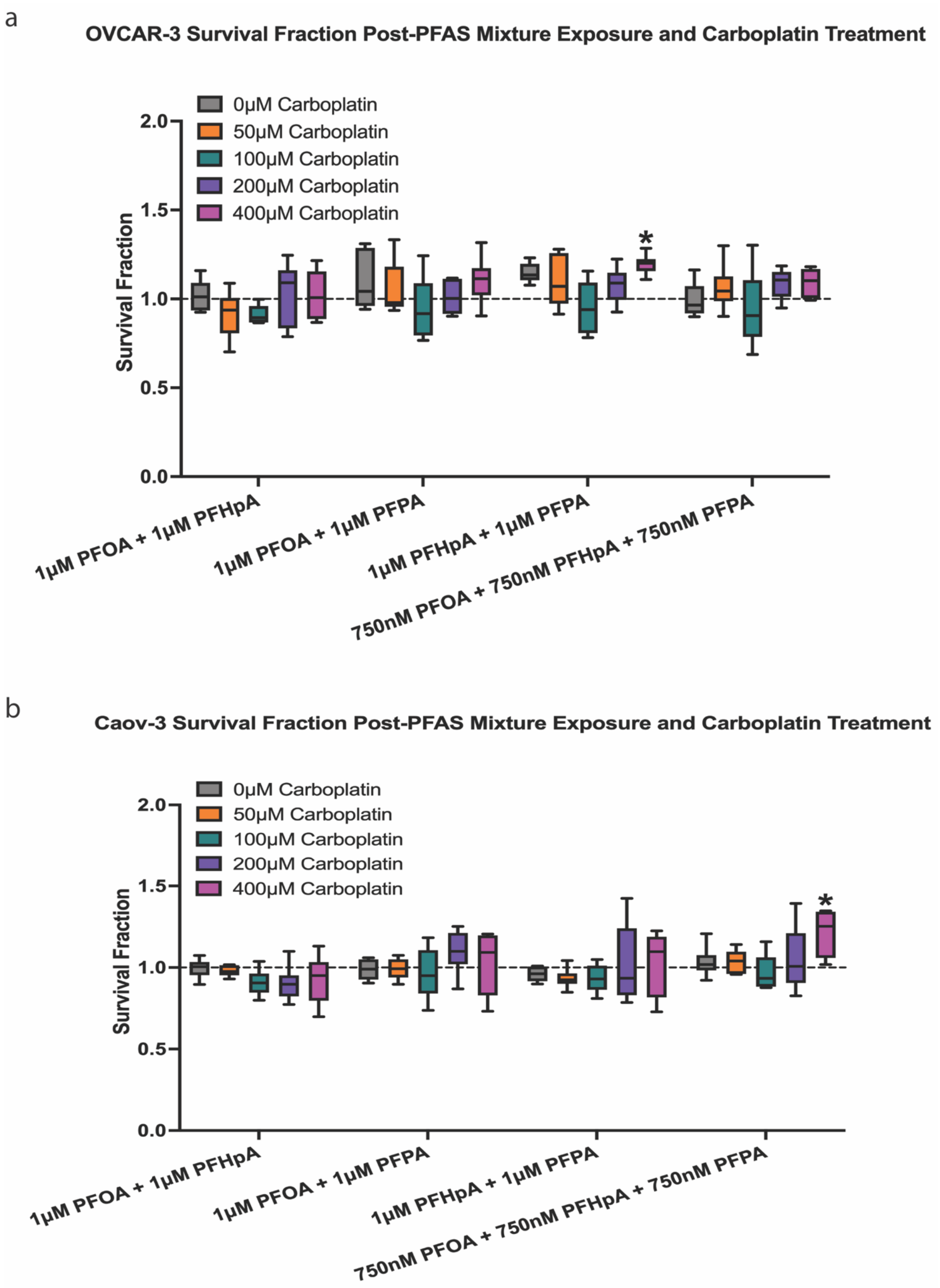

Figure S5. Select PFAS mixtures increase survival fraction post-carboplatin treatment in ovarian cancer cells. **(a)** OVCAR-3 cell survival fraction increases post-1μM PFHpA + 1μM PFPA and 400μM carboplatin treatment. **(b)** Caov-3 cell survival fraction increases post-750nM PFOA + 750nM PFHpA + 750nM PFPA and 400μM carboplatin treatment. OVCAR-3 and Caov-3 cell viability [mean ± SD expressed as a percentage of the vehicle control (dashed line) for each carboplatin group; *n* = 3 independent experiments in duplicate] after 48 h exposure to PFOA + PFHpA, PFOA + PFPA, PFHpA + PFPA or PFOA + PFHpA + PFPA followed by 48 h treatment with carboplatin.

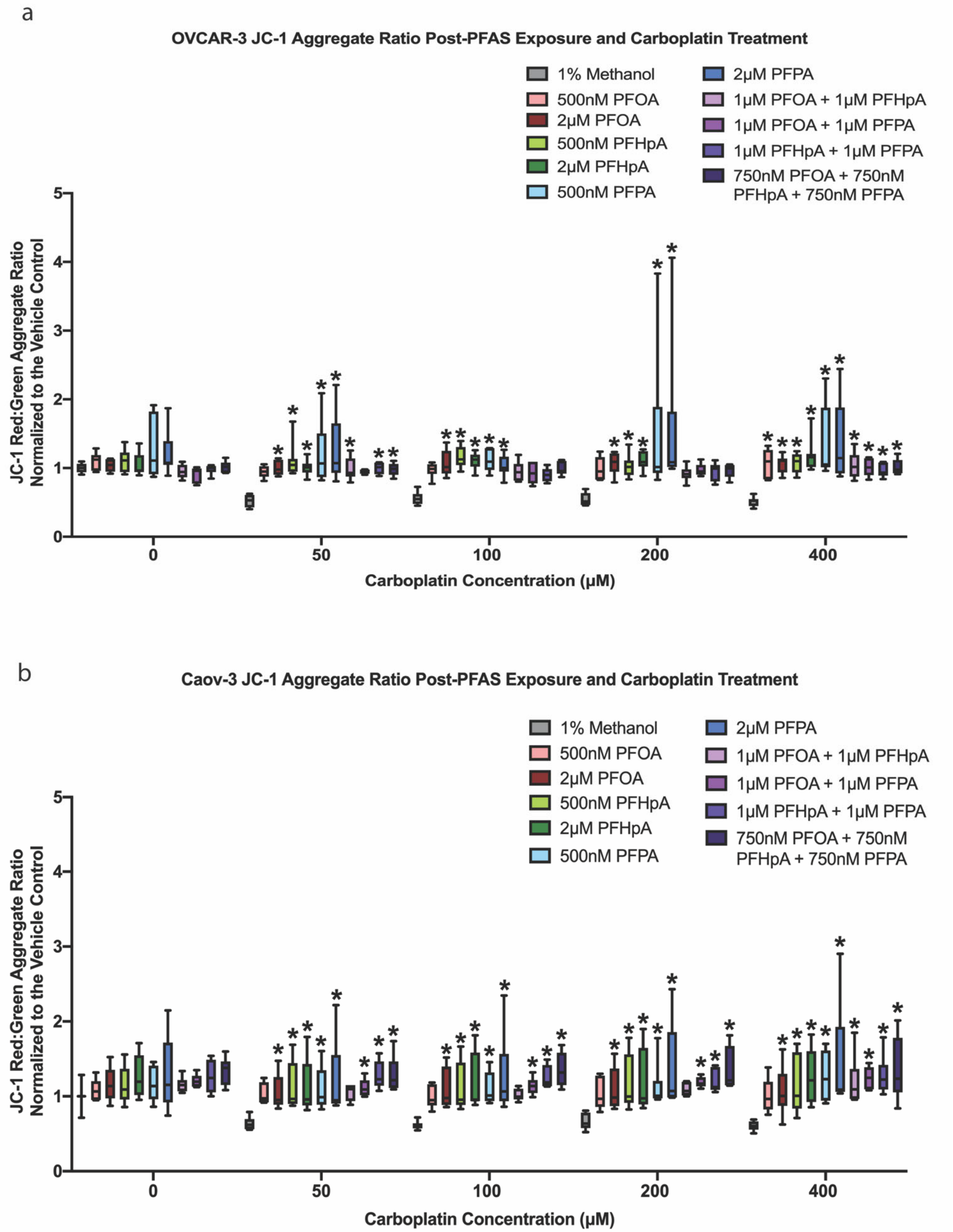

Figure S6. $\Delta\Psi_m$ increases post-PFAS exposure and carboplatin treatment in OVCAR-3 and Caov-3 cells. In **(a)** OVCAR-3 and **(b)** Caov-3 cells, $\Delta\Psi_m$ increases in every PFAS exposure group and at least one carboplatin concentration [mean ± SD expressed as a percentage of the vehicle control for each carboplatin group; n = 3 independent experiments in triplicate].

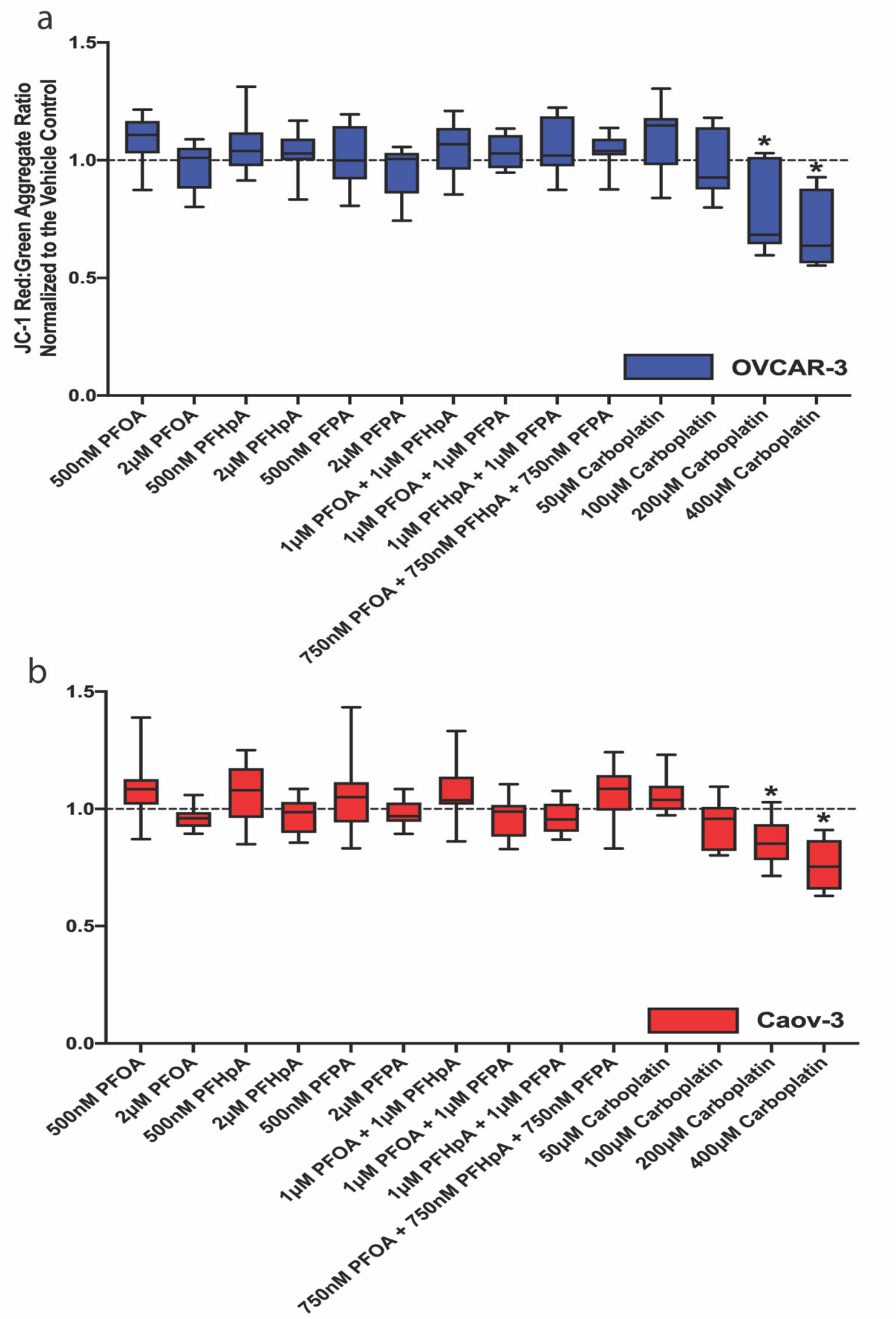

Figure S7. Carboplatin treatment, but not PFAS exposure, decreases $\Delta\Psi_m$ in ovarian cancer cells on experimental day 6. $\Delta\Psi_m$ decreases in **(a)** OVCAR-3 and **(b)** Caov-3 cells following 48-hour treatment with 200μM and 400μM carboplatin, but is unaffected by 48-hour PFAS or PFAS mixture exposure followed by a 48-hour incubation with fresh medium [mean ± SD expressed as a percentage of the vehicle control (dashed line); *n* = 4 independent experiments in triplicate].

Exposure Group by Cell Line (OVCAR-3 = blue-shaded region, Caov-3 = red-shaded region)	IC_{50} (µM carboplatin)
Vehicle Control (Figure S3A Internal)	58.42
500nM PFOA	61.72
2µM PFOA	58.26
500nM PFHpA	62.83
2µM PFHpA	71.49
500nM PFPA	46.96
2µM PFPA	63.47
Vehicle Control (Figure S4A Internal)	42.50
1µM PFOA + 1µM PFHpA	40.72
1µM PFOA + 1µM PFPA	44.28
1µM PFHpA + 1µM PFPA	41.62
1µM PFOA + 1µM PFHpA + 1µM PFPA	46.02
Vehicle Control (Figure S3B Internal)	164.5
500nM PFOA	179.3
2µM PFOA	169.2
500nM PFHpA	189.1
2µM PFHpA	193.9
500nM PFPA	150.9
2µM PFPA	163.1
Vehicle Control (Figure S4B Internal)	116.0
1µM PFOA + 1µM PFHpA	119.6
1µM PFOA + 1µM PFPA	135.9
1µM PFHpA + 1µM PFPA	130.9
1µM PFOA + 1µM PFHpA + 1µM PFPA	128.8

Table S1. OVCAR-3 and Caov-3 IC_{50} values post-PFAS or PFAS mixture exposure and carboplatin treatment. IC_{50} values for OVCAR-3 cells (blue-shaded region) and Caov-3 cells (red-shaded region) post-PFAS or PFAS mixture exposure and carboplatin treatment determined using nonlinear regression analysis. Each PFAS exposure group was internally normalized to its own vehicle control which is appropriately labeled for accurate comparison.
